# Computer-aided transrectal ultrasound: does prostate HistoScanning™ improve detection performance of prostate cancer in repeat biopsies?

**DOI:** 10.1186/s12894-015-0072-z

**Published:** 2015-07-30

**Authors:** Moritz Franz Hamann, C. Hamann, A. Trettel, K P Jünemann, C M Naumann

**Affiliations:** Department of Urology and Pediatric Urology, University Hospital Schleswig Holstein, Campus Kiel, Arnold Heller Str. 3, 24105 Kiel, Germany

**Keywords:** Prostate, Prostate cancer, Transrectal ultrasonography, HistoScanning, Prostate biopsy

## Abstract

**Background:**

An imaging tool providing reliable prostate cancer (PCa) detection and localization is necessary to improve common diagnostic pathway with ultrasound targeted biopsies. To determine the performance of transrectal ultrasound (TRUS) augmented by prostate HistoScanning^TM^ analysis (PHS) we investigated the detection of prostate cancer (PCa) foci in repeat prostate biopsies (Bx).

**Methods:**

97 men with a mean age of 66.2 (44 – 82) years underwent PHS augmented TRUS analysis prior to a repeat Bx. Three PHS positive foci were defined in accordance with 6 bilateral prostatic sectors. Targeted Bx (tBx) limited to PHS positive foci and a systematic 14-core backup Bx (sBx) were taken. Results were correlated to biopsy outcome. Sensitivity, specificity, predictive accuracy, negative predictive value (NPV) and positive predictive value (PPV) were calculated.

**Results:**

PCa was found in 31 of 97 (32 %) patients. Detection rate in tBx was significantly higher (p < .001). Detection rate in tBx and sBx did not differ on patient level(p ≥ 0.7). PHS sensitivity, specificity, predictive accuracy, PPV and NPV were 45 %, 83 %, 80 %, 19 % and 95 %, respectively.

**Conclusions:**

PHS augmented TRUS identifies abnormal prostatic tissue. Although sensitivity and PPV for PCa are low, PHS information facilitates Bx targeting to vulnerable foci and results in a higher cancer detection rate. PHS targeted Bx should be considered in patients at persistent risk of PCa.

## Background

Transrectal ultrasound (TRUS) imaging and systematic TRUS-guided biopsies (Bx) are gold standard procedures for prostate diagnostics and detection of prostate cancer (PCa) [1, 2]. However, the power to identify - and in particular to exclude - cancer reliably is limited due to low PCa specificity of grey scale ultrasound patterns [3]. Up to one-third of men with an initial negative systematic Bx are found to have prostate cancer in subsequent Bx [3, 4]. The question whether to pursue further repeat TRUS guided Bx in patients with a rising prostate-specific antigen (PSA) level subsequent to an initially negative Bx is a common clinical dilemma and remains a diagnostic challenge in urology. On the other hand, multiparametric magnetic resonance imaging (mp MRI) of the prostate is rapidly gaining significance due to its capability to detect PCa. Targeted biopsies of suspicious lesions, using MRI fused with real-time ultrasound, show encouraging rates of detection of clinically significant PCa [5]. In contrast to widely-used ultrasound, MRI hardware is expensive and time-consuming diagnostic protocols limit its availability. Further developments in ultrasound techniques, like contrast enhancement, elastography or prostate HistoScanning™ (PHS), improve TRUS capability to detect pathology confined to the prostatic gland and increase the validity of TRUS-guided Bx [6]. Initial data on PHS computer-aided ultrasonography have shown favorable results [7]. But evidence from existing Bx studies is scarce and controversial [8–11].

To generate a greater diagnostic yield than systematic needle Bx, we integrated the results of HS into our repeat prostate biopsy program. We report the cancer detection rate and characteristics of this approach in a prospective series of 97 consecutive patients with previous negative prostate Bx.

## Methods

At one center, data was collected from 97 consecutive men with a mean age of 66.2 (44 – 82) years [Table 1]. All of them were at a persistent risk of prostate cancer and had at least one previous set of TRUS-guided prostate Bx, yielding a non-cancerous diagnosis. All of them had suspicious findings at the digital rectal examination (DRE), or serum prostate-specific antigen (PSA) level >10 ng/mL, or both. Elevated serum PSA levels >4 ng/mL a PSA-velocity of >0.75 ng/mL p.a. and free-to-total PSA ratio < 15 % were seen as the indication for prostate biopsies. Rescanning of the prostate was scheduled at least three months after the previous manipulation of the gland in order to minimize impairment of data quality through earlier diagnostic procedures. All patients were informed of the mode of the extended prostate Bx scheme and its potential complications. All patients provided written informed consent for the procedures. Patients were advised that information collected from their Bx would be used for internal analysis and medical research as approved by the local Ethics Committee of the medical faculty of the University of Schleswig-Holstein, and was subsequently analyzed retrospectively.

**Table 1:** Patient characteristics

Mean (range) patient age	66.24 (44–82)
Mean (range) PSA level ng/mL	10.42 (1.02–35.00)
DRE pos / neg (%)	86/11 (89/11)
Prostate volume ml Mean (range)	51.43 (17.00/105.00)
Previous Biopsy sessions Mean (range)	1.46 (1–5)

### HistoScanning technique and prostate biopsy technique

After indication diagnostics and before starting the Bx procedure, all patients underwent an automatic standardized 3-dimensional (3D) transrectal ultrasound (TRUS) using a BK ProFocus UV ultrasound system with 8818 tri-plane probe (Analogic Corp, Peabody MA, USA). To facilitate appropriate data acquisition and a standardized scanning process, an external motor sweeps the ultrasound probe's sagittal array. Beside the signals from macroscopic tissue boundaries, which are used to create anatomical images, ultrasound produces a continuous stream of echoes emanating from the tissue`s underlying microscopic features, known as ultrasound backscatter. PHS analyses these backscatter signals by using numerical descriptors of multiparametric measures from the individual 3D raw data scan, which vary in its properties due to suspicious (malignant) or normal prostatic tissue characteristics. A statistical classifier categorizes corresponding prostatic regions as normal or suspicious. Displaying these informations as a colored (red) overlay in the ultrasound images complements the conventional ultrasound grey-scale diagnostics.

**Figure 1:**
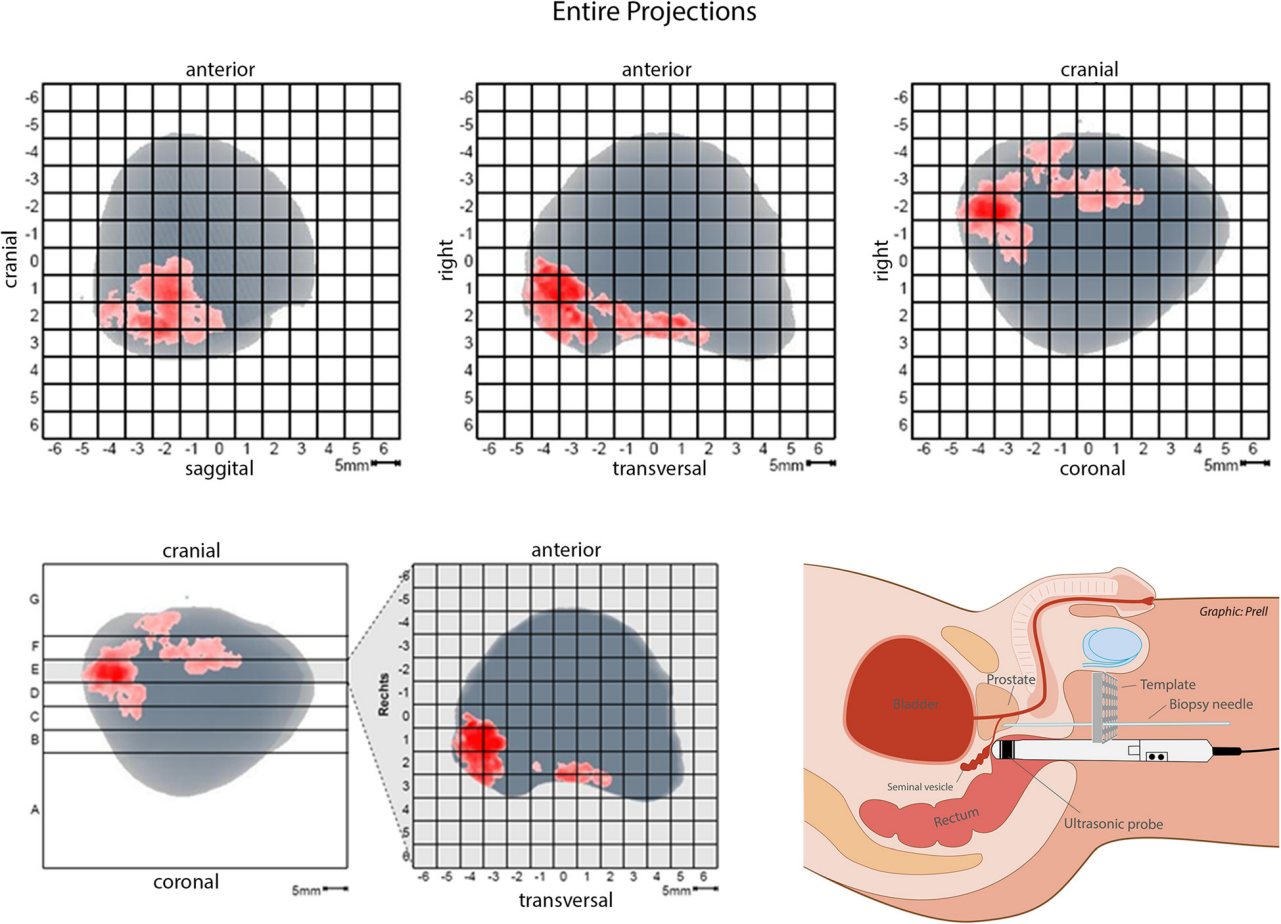
HistoScanning report and perineal biopsy setting PHS analysis provides a transversal slice report of the unique prostate showing suspicious areas with a spatial resolution of 5 mm. The report allows precise and stable constant targeting by the use of an adapted template grid fixed to a cradle

Computer-aided analysis of the data was performed on a PHS workstation with software version 2.3 (Advanced Medical Diagnostics, Waterloo BE). Based on the PHS image, the physician defined the most prominent (largest) target regions, up to a maximum of three. In turn, a structured scaffold was created from PHS projection reports of the prostate, which was used at a later point for guidance of targeted Bx procedures (Fig. 1). The location of the suspicious/target regions was defined according to 12 peripheral sectors and two central sectors: a bilateral transition zone, apex, center, and base (each of the latter three medially and laterally).

The biopsy procedures were performed consecutively during a single visit with the patient in a dorsal lithotomy position under general anesthesia. Three targeted cores were taken transperineally from each suspicious region based on PHS analysis (maximum of 3). A brachytherapy template grid fixed to a cradle was placed next to the perineum and used as a guide. Using the information from the PHS projection reports, the biopsy needle was directed through the brachytherapy template to obtain the Bx under direct TRUS guidance using a BK 8848 ultrasound probe (Analogic Corp, Peabody MA, USA). Sampling a target lesion, neither the needle position (grid perforation) nor the depth of the biopsies was changed (Fig. 1). Thereafter, three targeted cores were taken by a transrectal approach from each suspicious region using the tri-plane 8818 probe. Using the information from PHS analysis, Bx was directed cognitively to the PHS positive volumes. Finally, a standardized 14-core transrectal-guided Bx was performed by sampling the corresponding seven sectors bilaterally, as mentioned above. Biopsies were performed randomly by four senior surgeons with at least five years of biopsy experience.

The local pathologists of the University Hospital Schleswig Holstein, Campus Kiel, performed histopathology analysis of Bx cores.

### Statistical analysis

All data were registered in a Microsoft Access database (2010) and tabulated subsequently in Microsoft Excel (2010), with statistical analysis performed using PC SAS (Version 9.3 or higher) and R (version 3.0.1). For calculation on paired data the McNemar test was used. A two-sample Chi-squared test of proportions was used for binomial data. Differences in total PHS suspicious volume were compared using the Wilcoxon signed rank test. A p-value of 0.05 was considered to indicate statistical significance.

The cancer detection rate and procedure characteristics for all cases were evaluated. Table 1 summarizes the patient characteristics.

## Results

PHS analysis detected abnormalities in all prostate scans of the 97 patients included. 224 sectors contained PHS positive ultrasound patterns. In 31 of 97 (32 %) patients prostate adenocarcinoma was diagnosed, and 40 of 248 (16 %) sectors harbored cancer with respect to biopsy results. Additionally, histopathological examination detected atypical small acinar proliferation in seven patients (7 %), high-grade prostatic intraepithelial neoplasia in 20 (21 %) and chronic active inflammation in 72 (74 %) respectively. Based on the combined outcome, diagnostic performance of PHS to detect cancer at sector level shows sensitivity, specificity, predictive accuracy, a positive predictive value (PPV) and a negative predictive value (NPV) of 45 %, 83 %, 80 %, 19 % and 95 %, respectively. Table 2 summarizes the histological findings for all patients. The median PHS sum of the suspicious volume of 3 regions was 1.32 ml (range 0.20-9.32). Eighty seven patients (90 %) had a PHS sum ≥0.50 ml. Bx outcome and Gleason sum in relation to PHS suspicious volume is presented in Table 3. With respect to biopsy results, PHS volumes of benign prostate sectors and prostate sectors harboring cancer did not differ significantly. In 32 out of 77 (42 %) positive biopsies, the cancerous tissue was found in the anterior zone. 34 (44 %) respectively 21 (27 %) cancerous cores were harvested from the medial and basal parts of the gland. The occurrence of PHS positive ultrasound patterns did not differ significantly at sector level. Although the targeted biopsies originated from a maximum of three prostatic sectors, the detection rate per patient was not significantly different from systematic Bx. The detection rate for each PHS positive region was significantly higher in both targeted transperineal and targeted transrectal Bx compared to systematic Bx(p < .001). Table 4 shows an analysis of the detection rates of different procedures. Twenty two cancers were detected by systematic biopsies, while 20 and 21 cancers were detected in PHS targeted perineal and transrectal biopsies, respectively. The detection rate of significant cancer (GS ≥ 7) in PHS-targeted biopsies was superior in comparison to systematic biopsies. Perineal PHS targeted biopsies missed 1 significant cancer, PHS targeted transrectal biopsies missed 3 significant cancers, respectively. Most of the cancers (18/23, 78 %) detected by systematic biopsy were insignificant.

**Table 2:** Histopathological results in prostate biopsies

		Systematic	HS-template targeted transperineal	HS- targeted transrectal	All biopsies
Bx+		23	20	21	31
GS sum in Bx+	Mean (range)	6.5 (5–9)	6.5 (5–9)	6.38 (5–9)	6.35 (5–9)
GS sum ≥7 in Bx+	N (%)	5 (5)	10(10)	8(8)	11 (11)
GS I = 2/3 in Bx+	N (%)	19(19)	14(14)	16(16)	24 (25)
GS I = 4/5 in Bx+	N (%)	4(4)	6(6)	5(5)	7 (7)
ASAP	N (%)	3(3)	2(2)	2(2)	7 (7)
HG-PIN positive	N (%)	12(12)	9(9)	12(12)	20 (21)
Prostatitis	N (%)	68(70)	59(61)	59(61)	72 (74)

Bx+, prostate cancer positive biopsy; GS, Gleason Score; ASAP, atypical small acinar proliferations; HG PIN, high-grade intra epithelial proliferations

**Table 3:** Relation between Prostate HistoScanning™ suspicious volume and Bx outcome

		Bx +	Bx-	p-value
Total PHS suspicious volume in ml	N	31	66	
	median(range)	1.58 (0.20-9.32)	1.26 (0.22-6.59)	n.s.
		Gleason sum <7	Gleason sum > =7	
Total PHS suspicious volume in ml	N	20	11	
	median (range)	1.55 (0.20-9.32)	1.86 (0.22-4.35)	n.s.

PHS, prostate HistoScanning; Bx, prostate biopsy; Bx+, prostate cancer positive biopsy; Bx-, prostate cancer negative biopsy; p-values Bx + vs Bx- (Wilcoxon rang sum test)

**Table 4:** Analysis of detection rate per prostate region of different procedures

N = 97	Systematic Bx	PHS-template targeted transperineal Bx		PHS- targeted transrectal Bx	
patients with Bx+	23	20	n.s.	21	n.s.
regions biopsied	1358	248		248	
regions with Bx+	73	32		28	
detection rate per region	5 %	13 %	<0.001	11 %	<0.001

Biopsy Bx, prostate cancer positive biopsy Bx+, Pca negative biopsy Bx-, p-values compared with systematic biopsy (Chi-squared test)

## Discussion

Cancer specific accuracy in standard prostate Bx tends to be determined by PSA, prostate texture recorded by digital rectal examination as well as the number of biopsy cores in relation to prostate size, rather than by transrectal grey scale ultrasound displaying malignant foci [12]. Thus, a substantial number of patients undergo repeat biopsy, indicated by weak prognostic factors, still facing the same limited cancer-specific imaging characteristics of TRUS. With respect to initial data, PHS seems to overcome these limitations by analyzing radiofrequency backscattered raw data of prostatic ultrasound [13]. Our data is unable to confirm a comparable high prognostic validity for prostate cancer. In the present study, PHS revealed at least 2 to 3 aberrations in 94 % and 59 % of the ultrasound scans, but histopathological analysis confirms malignant tissue in only 32 % of the patients. Although, these detection results are within the range of previous studies on repeat biopsies [4], calculations of PHS predictive characteristics show weak results with respect to PPV (17 %) and sensitivity (44 %). These findings are in line with recent studies which also failed to show significant accuracy for PHS to predict positive biopsy results: According to sextant analysis, PHS had a sensitivity, specificity, PPV and NPV of 32.0-100 %, 5.9-32.7 %, 19.6-22.7 % and 86.4-84 %, respectively [9, 10]. Further analysis found no correlation between pathology and the whole gland tumor volumes estimated by PHS, nor between the PHS volumes and prostate cancer GS [10, 14]. Table 3 shows comparable results. It has to be taken into account, that PHS algorithms allow exclusively two opposed classifications (suspicious vs. non-suspicious) for ultrasound properties in virtual subvolumes. Based on a repeated but uniform calculation, the number of decisions as displayed by the PHS volume does not display varying characteristics or allow risk stratification concerning Gleason grading. Additionally, it must be assumed that acoustic similarity in the ultrasound backscatter properties of malignant and nonmalignant structures might account for further limitations in the analysis. With respect to specificity and NPV of 83 % and 95 %, respectively, PHS results need careful interpretation before the results can make a contribution to decision-making like biopsy indication or planning.

On the other hand, the detection rate in our cohort improved significantly by PHS-targeted biopsies. On patient level, the PHS template-targeted biopsies account for a 27 % increase in the detection rate, transrectal-targeted biopsies for 23 %, and the combined targeted biopsies for an increase by 41 % compared to systematic biopsy alone. PHS perineal targeted biopsies detect twice as much significant cancer foci than systematic ultrasound-guided biopsies. De Coninck et al. reported comparable findings in 41 men who underwent prostate biopsies supplemented with cognitive-targeted biopsies in suspicious regions based on prostate PHS. Targeted biopsies were 4.5-fold more likely to be positive than the random biopsies [11]. In our series, the rate of non-significant cancers detected by PHS-targeted perineal vs. systematic Bx was lower (Table 2). This rebuts potential concerns of an increased detection of clinically insignificant cancers caused by more substantial sampling. PHS-targeted biopsies detected 6 significant prostate cancers that were overseen by systematic biopsy. Additionally, the absolute numbers of atypical histopathological findings like ASAP, HG PIN and prostatitis in PHS-positive sectors do not differ significantly from systematic Bx despite the limited sampling.

Our data might suggest a reduction of biopsies in non-PHS positive sectors and to focus on more vulnerable or suspect regions in the prostate. The corresponding PB scheme would theoretically achieve appropriate sampling by targeting certain locations rather than by increasing the core numbers. In an analysis of 164 autopsies, which had not previously undergone clinical biopsy, step-section analysis revealed that the common 12-core biopsy technique detects the majority of clinically significant cancers with a sensitivity of 80 % [15]. The authors conclude that the chance of detecting cancer was correlated more closely to the location of the sampling (peripheral, lateral and apical cores) than to the number of biopsy cores taken.

Prostate cancer detection rates from perineal targeted biopsies differed from the transrectal targeted biopsy regime. This difference occurred independently from previous tissue analysis, for the reason that both targeted approaches are aligned to the same scanning process. In comparison to the transrectal approach, the perineal biopsy technique might reduce variables that can influence the needle placement. Furthermore, longitudinal biopsy punches following the axis of the prostate seem to allow more accurate sampling of the anterior part. Theoretically, because previous studies reported inhomogeneous results comparing transrectal and transperineal prostate biopsies [8, 16].

There are several limitations in this study. Firstly, the analysis was performed on a regional basis. Dependencies between neighboring regions, as well as correlation of the different biopsy procedures with the HS results are difficult to account for and may incur misleading results. Secondly, there is no minimum threshold defining when a PHS signal is to be called positive; this may also affect the results. For example, the targeted regions could stretch across different sections meaning that even a small amount in one sector would lead to it being seen as a positive PHS sector, but it might still yield negative biopsies because the few cores would not necessarily pick up the cancer. Moreover, the knowledge of “abnormal” regions might influence the surgeon’s decisions during the subsequent systematic biopsy as a result of the non-blinded, single-surgeon biopsy procedure. Nevertheless, we did not find any significant inter-operator variability with respect to the detection rate or the biopsy approach.

Generally, it is difficult to compare the diagnostic accuracy of Bx techniques in different patient series. Ethical concerns will always prevent verification of negative Bx results by radical prostatectomy, and positive biopsies are not always and necessarily followed by prostatectomy. Therefore, the clinical setting will always account for a potential verification bias, not knowing what the exact location of the cancer is and how many cancers were missed by biopsy.

Finally, recent results from multi-parametric MRI procedures pull into question all ultrasound-based techniques including color Doppler imaging, real-time elastography, C-TRUS / ANNA and Prostate HS. The application of integrated interpretation strategies for mp MRI variables, such as the PIRADS score, improves the risk stratification for prostate cancer and allows for effective fusion guidance of targeted prostate biopsy [17, 18]. Likewise, all ultrasound modalities have been devised to increase the diagnostic yield of prostate biopsy, using ultrasound data to visualize and locate tissues suspected of harboring prostate cancer [19–21]. Despite their promising characteristics, none of them are established in clinical practice because of missing evidence, lack of standardization and significant user-dependent performance. Similarity in the prostate tissue features such as the degree of elasticity/firmness, irregular patterns of blood vessels and ultrasound backscatter properties of malignant and other non-malignant microstructures might account for these limitations. So far, each technique in itselfis limited in its predictive impact on prostate cancer diagnostics, but might be combined into an effective diagnostic tool. Accordingly, a multiparametric approach similar to MRI algorithms might improve future performance and increase the diagnostic yield by specifically visualizing and locating prostate cancer tissue.

## Conclusions

HS-augmented ultrasound analysis improves the interpretation of transrectal prostate imaging by identification of abnormal prostatic tissue. Although prostate cancer specificity remains low, the additional information facilitates improved biopsy targeting and results in a higher cancer detection rate in selective prostate sectors. The high NPV might help to reduce biopsies in PHS-negative prostate sectors and to focus on more vulnerable regions in the prostate. With the help of this strategy, increased but unsought detection of clinically insignificant cancers through too substantial sampling can be avoided. The PHS technique should be considered at leastwith respect to patients who had previously negative prostate biopsy results despite elevated PSA levels. Further multicenter analysis has to confirm the advantages of PHS in primary Bx-settings.

## Abbreviations

TRUS: Transrectal ultrasound
PHS: Prostate HistoScanning™
PCa: Prostate cancer
Bx: Prostate biopsies
tBx: Targeted prostate biopsies
sBx: Systematic prostate biopsies
NPV: Negative predictive value
PPV: Positive predictive value
PSA: Prostate specific antigen
mp MRI: Multiparametric magnet resonance imaging
